# ERAP1 Gene Variants and Haplotypes Associated With Psoriasis Vulgaris of Han Chinese in Inner Mongolia

**DOI:** 10.1002/mgg3.70021

**Published:** 2024-11-21

**Authors:** Xin Li, Jia Bao, Liya Ai, Fan‐Rui Yang, Bo Yu, Yan‐Ping Huang, Na Li, Wen‐Yuan Ding, Zhi‐Qiang Sun, Xin‐Xiang Lv, Jian‐Wen Han

**Affiliations:** ^1^ Department of Dermatology The Affiliated Hospital of Inner Mongolia Medical University Hohhot Inner Mongolia China

**Keywords:** association, ERAP1, genetic variation, HLA‐C, psoriasis

## Abstract

**Background:**

This study aimed to investigate the association between genetic variants of *ERAP1* (OMIM: 606832) and psoriasis vulgaris (PsV) susceptibility in Inner Mongolia Han nationality.

**Methods:**

For primary screening, the subjects included 142 PsV cases and 100 healthy controls without psoriasis. The 27 exons of *ERAP1* gene were sequenced to screen significant genetic variants. For the validation study, the subjects included 1030 PsV cases and 965 healthy controls. A total of 18 mutations were detected for genetic variants of significance in primary screening and previously reported genetic variants.

**Results:**

In primary screening stage, 13 genetic variants of *ERAP1* showed an association with psoriasis. A total of 18 genetic variants were typed for the validation, and 12 genetic variants were associated with PsV in Inner Mongolia Han population. Stratified analysis showed significant differences in the allele frequencies of 8 *ERAP1* genetic variants in cases with positive family history, and significant differences in allele frequencies among 9 *ERAP1* genetic variants in patients with negative family history. A risk haplotype (TCCCTCCAGACC) was significantly associated with PsV, and the most risk haplotype was E730/K528/R127/E56.

**Conclusion:**

*ERAP1* gene mutation may be associated with PsV and HLA‐C*06:02 in Han nationality in Inner Mongolia. A risk haplotype of four‐nonsynonymous mutation (E730/K528/R127/E56) is associated with PsV.

## Graphical abstract

*ERAP1* gene mutation may be associated with psoriasis vulgaris and HLA‐C0602 in Han nationality in Inner Mongolia. A risk haplotype of four‐nonsynonymous mutation (E730/K528/R127/E56) is associated with PsV.

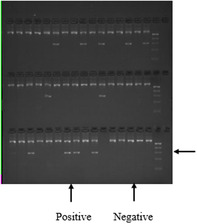

## 1 Introduction

Psoriasis vulgaris (PsV) is a chronic inflammatory skin disease characterized by skin erythema and scaling, with a prevalence of about 0.1%–2.8% of the total population worldwide and an overall prevalence of about 0.47% in China (Xiaolan et al. 2010). According to the age of onset, PsV can be divided into early‐onset PsV (age of onset ≤ 40 years) and late‐onset PsV (age of onset > 40 years), and the incidence of early‐onset PsV is higher in the PsV family (Fanoni et al. 2019; Guo et al. 2023; Szentkereszty‐Kovács et al. 2019). It is well‐known that *ERAP1* gene (OMIM: 606832) is involved in PsV, and Tang et al. conducted a genetic study of 10,727 PsV cases and 10,582 controls in China, and the results showed that rs27044 (Q730E) and rs26653 (R127P) on *ERAP1* were significantly associated with PsV (Tang et al. 2014). A European study showed that rs27524A at the *ERAP1* variant was associated with psoriasis (*p* = 0.019, OR = 1.34), especially in pediatric patients, which could be used as an independent risk factor (*p* = 0.042, OR = 1.43) (Bergboer et al. 2012). Therefore, *ERAP1* is highly polymorphic and may be involved in the pathogenesis of PsV.

Human leukocyte antigen (*HLA*) (OMIM: 142840) is PsV‐associated gene, especially for HLA‐C*06:02 (Zhen et al. 2019). Previous studies suggest that *ERAP1* and HLA‐C*06:02 interact in the pathogenesis of PsV. Das et al. (2017) analyzed the relationship between *ERAP1*, HLA‐C*06:02, and PsV in an eastern Indian population and found that three genetic variants of *ERAP1* (rs27044, rs30187, rs26653) were associated with PsV only when HLA‐C*06:02 was positive. Lysell et al. (2013) reported that the association between rs26653 on the *ERAP1* gene and PsV was not limited to HLA‐C*06:02 positive individuals. There may be an interaction between *ERAP1* and HLA‐C*06:02, but due to ethnic and geographical differences, the results are not consistent and there may be genetic heterogeneity.

It has been previously reported in China that *ERAP1* genetic variants rs27044, rs26653, rs30187, rs27524, and rs151823 are associated with PsV (Shahid et al. 2023; Yang et al. 2013), but the mechanism of interaction between *ERAP1* and HLA‐C*06:02 has not been fully elucidated, and there are ethnic and regional differences in the genes. In order to clarify the association between *ERAP1* gene variants and PsV in Inner Mongolia Han nationality, and the effects of these genetic variants and HLA‐C*06:02 interaction on PsV susceptibility in Inner Mongolia Han nationality, we performed this study with a large sample size and carried out genetic association analysis studies using a two‐stage experimental method of primary screening and validation.

## 2 Materials and Methods

### 2.1 Ethical Compliance

This study was approved by the Ethics Committee of Inner Mongolia Medical University and adhered to the Declaration of Helsinki during its implementation, and informed consent was obtained from all study subjects.

### 2.2 Subjects

The study population consisted of 1172 patients with PsV and 1065 healthy controls who visited the Affiliated Hospital of Inner Mongolia Medical University from December 2010 to March 2020. All subjects recruited were Han Chinese over 18 years of age, male or female. Of these, 142 PsV patients and 100 healthy controls served as primary screening sequencing samples. The remaining samples (1030 PsV patients and 965 healthy controls) were subjected to validation experiments. Patients with PsV were diagnosed by two chief physicians based on histopathology. Healthy controls with no personal history of PsV were recruited from the physical examination center of the hospital, and no PsV patients among the first‐, second‐, and third‐degree relatives. This study was approved by the Ethics Committee of Inner Mongolia Medical University and adhered to the Declaration of Helsinki during its implementation, and informed consent was obtained from all study subjects.

### 2.3 Next‐Generation Sequencing

Blood samples were collected from all subjects the next morning after fasting for 8 h for examination, and 4 mL of peripheral blood samples were collected from each subject and stored at −80°C for future use. Genomic DNA from blood samples was extracted using a DNA extraction kit (AxyPrep, AP‐MX‐BL‐GDNA‐25, Suzhou, China). Information on all SNPs was obtained from the PubMed database. The multiplex PCR reaction system included: 1 μL DNA template, 2 μL buffer, 0.6 μL Mg2+, 2 μL dNTPs, 0.2 μL Taq DNA polymerase, 2 μL primer mixture, supplemented with double‐distilled water. The primers are listed in Table 1. PCR reaction conditions: pre‐denaturation at 95°C for 2 min; 35 cycles of annealing at 94°C for 30 s, 56°C for 1.5 min, and extension at 65°C for 30 s; and finally extension at 65°C for 10 min.

**TABLE 1:** Primer sequence of 18 variants of ERAP1 in validated stage.

Gene	Single nucleotide polymorphism	Sense primer strand	Antisense primer strand
ERAP1	Rs27980	AATCAATATGAGAAGGAAATAGAAATAGC	CACAATGCCATCACTTAACTATAACTC
ERAP1	Rs27042	TACTGGGTTCCTGCCAATGAG	GCAGTGATTCATAACTGTTTCATCAC
ERAP1	Rs27044	CTCTGTACGCACGGCTGATAG	CTGTTTCCCTGTACAACGCC
ERAP1	Rs17482078	CTCTGTACGCACGGCTGATAG	CTGTTTCCCTGTACAACGCC
ERAP1	Rs469876	GCCTTAGTAGTCAGACAGGAGGT	TTCTGAAAGCAGCTGTTGTATTTC
ERAP1	Rs469783	AAACCGCGACTTTGTGCAG	TGGCTATTACGTGCATTACG
ERAP1	Rs469758	CCATTCCACCTCTTCTGGGAG	TTGAGGCTTTGTTTTCTGGTTTC
ERAP1	Rs10050860	TTCACATTCCTTGAATTAACTAG	GAATTGAATCAACAAATGAACAATC
ERAP1	Rs30187	CCGTCAGAGCCCTTCATGTAG	TGTGTACACACCAGCATTGGC
ERAP1	Rs26510	GCAGTCTTATCTGGAATGAGGGT	CTAGAAGTCAATTACCATCTTCATCC
ERAP1	Rs77311599	TTACAAACAATCATAGATTCAACTG	GAAAATATTCACTAATCTTTGATTATAGAAC
ERAP1	Rs27434	GCTTTGGAGAAACAATTTTTCCT	CATCAAGTAAGCTTGGCATCAC
ERAP1	Rs2287987	GCTTTGGAGAAACAATTTTTCCT	CATCAAGTAAGCTTGGCATCAC
ERAP1	Rs998509	CTCAGCCTCCCAAACTGTTG	AAGAGTGGAGTCAAGGTGAGCC
ERAP1	Rs766222717	CTCAAAATCTGAAATGATGAAGGC	GTTCACTGTATTGGATAATTGGATTAC
ERAP1	Rs26653	CCAGCATAGTGAATGACAACTGTG	GTCACCACCTGCAGATATCTAGG
ERAP1	Rs3734016	TCTACTTTCGTGGTTCCCCAG	TCCTGGTTCATGTCAGAGCAC
ERAP1	Rs151949	GCAGGCGGGGAAGCC	GTGCACTTCCTACGCCTGATC

Illumina second‐generation sequencing platform (Illumina Miseq) was used to sequence and analyze PCR products. The data analysis was performed to interpret the genotyping of each sample at each site. Sequence‐specific primer‐guided PCR reaction (PCR‐SSP) for HLA‐C*06:02 polymorphism (Gene accession No: OP585164.1). The primers are listed in Table 2. PCR amplification reaction: the total volume of the reaction was 25 μL, including PCR mixed buffer 12.5 μL, DNA model 3 μL, upstream primers 1 ul, downstream primers 1 μL, and double‐distilled water 7.5 μL. Reaction conditions: pre‐denaturation at 94°C for 180 s, denaturation at 94°C for 30 s, annealing at 60°C for 50 s, extension at 72°C for 60 s, and finally extension for 300 s for 35 cycles.

**TABLE 2:** Primer sequences of HLA‐C*06:02 gene.

Primer name	Upstream (5′‐3′)	Downstream (5′‐3′)	Length of PCR product
HLA‐C*06:02*	TACTACAACCAGAGCGAGGA	GGTCGCAGCCATACATCCA	297 bp
Internal control	GCATCTTGCTCTGTGCAGAT	TGCCAAGTGGAGCACCCAA	796 bp

*Note:* *Gene accession no: OP585164.1.

### 2.4 Statistical Analysis

Statistical analysis was performed using the PLINK1.07 software package (http://pngu.mgh.harvard.edu/purcell/plink/). The minimum allele frequency (MAF) of 27 exons in the control group was calculated, and Hardy–Weinberg equilibrium (HWE) test was performed. The MAF of each genetic variant locus was compared between the two groups by chi‐squared test, and the odds ratio (OR) of the minimum allele and its 95% confidence interval (95% CI) were calculated. Cochran–Mantel–Haenszel (CMH) method was used for the pooled analysis of data at the primary screening stage and the validation stage. For the validation stage classification data, stratified analysis was performed according to the age of onset, family history of PsV, and HLA‐C*06:02, and chi‐square tests were performed. *p* < 0.05 was considered statistically significant.

## 3 Results

### 3.1 Primary Screening

In the primary screening stage, we sequenced 27 exons of the *ERAP1* gene in 142 PsV vulgaris cases and 100 healthy controls, and a total of 157 genetic variants were found by sequencing. After quality control (removal of genetic variants that do not conform to HWE balance and removal of genetic variants with allele frequencies < 0.01), a total of 85 genetic variants were included in the statistical analysis. Statistical analysis showed that 13 genetic variants were associated with PsV (*p* < 0.05), including rs27980, rs27042, rs27044, rs469876, rs469783, rs469758, rs26510, rs27434, rs998509, rs766222717, rs3734016, rs151949, and rs77311599 (Table 3).

**TABLE 3:** Association of 18 ERAP1 variants with psoriasis in screening and validated stage.

SNP	BP	Allele	Preliminary screening test (142 cases and 100 controls)						Validation experiments (1030 cases and 965 controls)					
			MAF		*χ* ^2^	*p*	OR	95% CI	MAF		*χ* ^2^	*p*	OR	95% CI
			Case	Control					Case	Control				
rs27980	96762191	G	0.183	0.330	13.740	2.10E‐04	0.46	0.30–0.69	0.465	0.500	4.407	0.036	0.87	0.76–0.99
rs27042	96781236	A	0.134	0.225	6.870	0.009	0.53	0.33–0.86	0.264	0.272	0.237	0.626	0.96	0.83–1.12
rs27044	96783148	G	0.616	0.510	5.407	0.020	1.54	1.08–2.22	0.479	0.440	5.594	0.018	1.17	1.03–1.33
rs17482078	96783162	T	0.063	0.030	2.775	0.096	2.19	0.85–5.61	0.058	0.054	0.161	0.688	1.06	0.80–1.40
rs469876	96785702	G	0.053	0.165	16.530	4.78E‐05	0.28	0.15–0.54	0.289	0.313	2.615	0.106	0.89	0.77–1.03
rs469783	96785820	C	0.722	0.585	9.867	1.68E‐03	1.85	1.25–2.70	0.518	0.462	11.610	0.001	1.25	1.10–1.43
rs469758	96786011	C	0.884	0.675	31.680	1.81E‐08	3.70	1.25–2.70	0.513	0.463	9.091	0.003	1.22	1.07–1.40
rs10050860	96786506	T	0.077	0.050	1.434	0.231	1.60	0.74–3.45	0.028	0.025	0.271	0.603	1.12	0.73–1.73
rs30187	96788627	T	0.599	0.510	3.742	0.053	1.42	0.99–2.08	0.510	0.459	9.646	0.002	1.23	1.08–1.40
rs26510	96790207	C	0.778	0.575	22.820	1.78E‐06	2.56	1.75–3.85	0.511	0.459	9.870	0.002	1.23	1.08–1.40
rs77311599	96793259	T	0.021	0.055	3.973	0.046	0.37	0.13–1.02	0.026	0.038	4.354	0.037	0.67	0.46–0.98
rs27434	96793809	A	0.648	0.540	5.708	0.017	1.56	1.08–2.27	0.498	0.457	6.111	0.013	1.18	1.04–1.34
rs2287987	96793832	C	0.060	0.030	2.312	0.128	2.06	0.80–5.32	0.057	0.055	0.134	0.715	1.05	0.80–1.40
rs998509	96797092	A	0.063	0.145	8.917	2.83E‐03	0.40	0.21–0.74	0.141	0.168	5.060	0.024	0.81	0.68–0.97
rs766222717	96797330	C	0.081	0.145	5.015	0.025	0.52	0.29–0.93	0.135	0.166	6.987	0.008	0.78	0.65–0.94
rs26653	96803547	G	0.218	0.175	1.374	0.241	1.32	0.83–2.08	0.465	0.500	4.371	0.037	0.87	0.76–0.99
rs3734016	96803761	T	0.085	0.145	4.404	0.036	0.54	0.31–0.97	0.132	0.168	9.002	0.003	0.76	0.63–0.91
rs151949	96808142	T	0.113	0.215	9.383	2.19E‐03	0.46	0.28–0.76	0.280	0.278	0.008	0.928	1.01	0.87–1.16

*Note:* Case indicates the case group; Control indicates the control group.

Abbreviations: Allele, allele; BP, physical locus; MAF, minimum allele frequency; SNP, single nucleotide polymorphism.

### 3.2 Validation of the Association of *ERAP1* With PsV

For validation, 1030 PsV cases and 965 healthy control samples were selected for second‐generation sequencing. A total of 18 genetic variants were selected, including 13 statistically significant sites in the primary screening stage, and five sites previously reported to be associated with PsV rs30187, rs17482078, rs2287987, rs10050860, and rs26653 (*p* > 0.05 in the primary screening stage). After quality control, 12 loci showed correlation with PsV, including rs27980, rs27044, rs469758, rs30187, rs26510, rs77311599, rs27434, rs998509, rs766222717, rs26653, rs3734016, and rs469783 (*p* < 0.05) (Table 3).

Among the loci analyzed, protective factors for the onset of PsV were rs27980_G (OR = 0.82), rs469876_G (OR = 0.84), rs77311599_T (OR = 0.63), rs998509_A (OR = 0.77), rs766222717_C (OR = 0.75), and rs3734016_T (OR = 0.73); the risk factors for the onset of PsV were rs27044_G (OR = 1.20), rs469783_C (OR = 1.32), rs469758_C (OR = 1.33), rs26510_C (OR = 1.33), rs30187_T (OR = 1.25), and rs27434_A (OR = 1.22). However, rs27042_A, rs2287987_C, rs17482078_T, rs26653_G, rs10050860_T, and rs151949_T were not significantly associated with PsV (*p* > 0.05).

Pooled analysis using CMH method for data in the primary screening stage and validation stage showed 12 significant *ERAP1* gene variants, which were rs27980, rs27044, rs469758, rs30187, rs26510, rs77311599, rs27434, rs998509, rs766222717, rs3734016, rs974683, and rs469876 (*p* < 0.05) (Table 4).

**TABLE 4:** Combined association analysis of 18 ERAP1 variants in screening and validated stages.

SNP	BP	Allele	MAF	*χ* ^2^	*p*	OR	95% CI
rs27980	96762191	G	0.455	9.522	0.002	0.82	0.73–0.93
rs27042	96781236	A	0.257	1.458	0.227	0.92	0.80–1.06
rs27044	96783148	G	0.472	8.850	0.003	1.20	1.07–1.36
rs17482078	96783162	T	0.055	0.847	0.358	1.13	0.87–1.48
rs469876	96785702	G	0.278	6.181	0.013	0.84	0.73–0.96
rs469783	96785820	C	0.510	17.710	2.57E‐05	1.32	1.15–1.47
rs469758	96786011	C	0.520	19.820	8.50E‐06	1.33	1.17–1.51
rs10050860	96786506	T	0.032	1.131	0.288	1.23	0.84–1.78
rs30187	96788627	T	0.492	12.560	3.94E‐04	1.25	1.10–1.41
rs26510	96790207	C	0.510	19.830	8.45E‐06	1.33	1.16–1.49
rs77311599	96793259	T	0.033	6.985	0.008	0.63	0.44–0.89
rs27434	96793809	A	0.491	9.598	0.002	1.22	1.07–1.37
rs2287987	96793832	C	0.055	0.695	0.404	1.12	0.86–1.47
rs998509	96797092	A	0.148	9.076	0.003	0.77	0.65–0.91
rs766222717	96797330	C	0.146	10.260	0.001	0.75	0.63–0.90
rs26653	96803547	G	0.450	2.733	0.098	0.90	0.79–1.02
rs3734016	96803761	T	0.146	12.290	4.56E‐04	0.73	0.62–0.87
rs151949	96808142	T	0.265	0.559	0.455	0.95	0.83–1.09

Abbreviations: Allele, allele; BP, physical locus; MAF, minimum allele frequency; SNP, single nucleotide polymorphism.

### 3.3 Stratified Analysis of the Association Between *ERAP1* and PsV

First, we used HLA‐C*06:02 for stratified analysis of the association between *ERAP1* gene variants and PsV in the Inner Mongolia Han population. When HLA‐C*06:02 was positive, there were 547 PsV cases and 165 healthy controls; when HLA‐C*06:02 was negative, there were 336 PsV cases and 790 healthy controls. The results showed that when HLA‐C*06:02 was positive, rs469783_C (*p* = 0.036, OR = 1.29), rs469758_C (*p* = 0.030, OR = 1.31), and rs26510_C (*p* = 0.047, OR = 1.28) were significantly associated with PsV, and there was no significant association when HLA‐C*06:02 was negative (Table 5).

**TABLE 5:** Association of 18 ERAP1 variants with PsV stratified by HLA‐C*06:02.

SNP	BP	Allele	HLA‐C*06:02 positive (547 cases and 165 controls)						HLA‐C*06:02 negative (336 cases and 790 controls)					
			MAF		*χ* ^2^	*p*	OR	95% CI	MAF		*χ* ^2^	*p*	OR	95% CI
			Case	Control					Case	Control				
rs27980	96762191	G	0.453	0.503	2.486	0.115	0.82	0.64–1.05	0.481	0.499	0.550	0.459	0.93	0.78–1.12
rs27042	96781236	A	0.252	0.282	1.186	0.276	0.86	0.65–1.13	0.283	0.269	0.437	0.508	1.07	0.87–1.31
rs27044	96783148	G	0.494	0.439	3.011	0.083	1.25	0.97–1.60	0.460	0.442	0.556	0.456	1.07	0.89–1.29
rs17482078	96783162	T	0.059	0.064	0.080	0.778	0.93	0.56–1.55	0.054	0.052	0.030	0.864	1.04	0.69–1.55
rs469876	96785702	G	0.268	0.304	1.611	0.204	0.84	0.64–1.10	0.317	0.315	0.006	0.937	1.01	0.83–1.23
rs469783	96785820	C	0.529	0.463	4.384	0.036	1.29	1.02–1.67	0.505	0.463	3.201	0.074	1.18	0.98–1.42
rs469758	96786011	C	0.526	0.457	4.693	0.030	1.31	1.03–1.69	0.499	0.466	1.953	0.162	1.14	0.95–1.37
rs10050860	96786506	T	0.025	0.033	0.500	0.479	0.76	0.36–1.62	0.033	0.023	1.645	0.200	1.46	0.82–2.59
rs30187	96788627	T	0.520	0.461	3.600	0.058	1.26	0.99–1.61	0.497	0.459	2.658	0.103	1.16	0.97–1.39
rs26510	96790207	C	0.523	0.461	3.930	0.047	1.28	1.01 to 1.63	0.496	0.460	2.359	0.125	1.15	0.96–1.38
rs77311599	96793259	T	0.029	0.045	2.033	0.154	0.64	0.34–1.19	0.021	0.037	3.835	0.050	0.56	0.31–1.01
rs27434	96793809	A	0.510	0.454	3.052	0.081	1.25	0.97–1.60	0.485	0.458	1.262	0.261	1.11	0.92–1.33
rs2287987	96793832	C	0.059	0.067	0.235	0.628	0.88	0.54–1.46	0.054	0.052	0.041	0.840	1.04	0.69–1.57
rs998509	96797092	A	0.136	0.146	0.172	0.678	0.93	0.65–1.32	0.146	0.172	2.343	0.126	0.82	0.64–1.06
rs766222717	96797330	C	0.132	0.146	0.382	0.537	0.89	0.63–1.27	0.137	0.170	3.758	0.053	0.77	0.60–1.00
rs26653	96803547	G	0.450	0.488	1.487	0.223	0.86	0.67–1.10	0.485	0.500	0.432	0.511	0.94	0.78–1.13
rs3734016	96803761	T	0.127	0.146	0.791	0.374	0.85	0.60–1.21	0.139	0.172	3.758	0.053	0.78	0.60–1.00
rs151949	96808142	T	0.268	0.295	0.865	0.352	0.88	0.67–1.16	0.296	0.275	0.976	0.323	1.11	0.90–1.36

*Note:* Case indicates the case group; Control indicates the control group.

Abbreviations: Allele, allele; BP, physical locus; MAF, minimum allele frequency; SNP, single nucleotide polymorphism.

Next, we focused on family history of PsV (122 cases with positive family history and 766 cases with negative family history), and the results showed that there were significant differences in the allele frequencies of eight *ERAP1* genetic variants in patients with positive family history compared to control group: rs469783, rs469758, rs30187, rs26510, rs27434, rs998509, rs944222717, and rs3734016 (*p* < 0.05). There were significant differences in allele frequencies among nine *ERAP1* genetic variants in patients with negative family history compared to control group: rs27044, rs469783, rs469758, rs30187, rs26510, rs77311599, rs27434, rs766222717, and rs3734016 (*p* < 0.05) (Table 6).

**TABLE 6:** Association of 18 ERAP1 variants with PsV stratified by family history.

SNP	BP	Allele	Family history of psoriasis (122 cases and 965 controls)						No family history of psoriasis (766 cases and 965 controls)					
			MAF		*χ* ^2^	*p*	OR	95% CI	MAF		*χ* ^2^	*p*	OR	95% CI
			Case	Control					Case	Control				
rs27980	96762191	G	0.454	0.500	1.765	0.184	0.83	0.64–1.09	0.466	0.500	3.673	0.055	0.88	0.76–1.00
rs27042	96781236	A	0.281	0.272	0.097	0.756	1.05	0.78–1.41	0.262	0.272	0.412	0.521	0.95	0.82–1.11
rs27044	96783148	G	0.500	0.440	3.123	0.077	1.27	0.97–1.66	0.476	0.440	4.316	0.038	1.15	1.01 to 1.32
rs17482078	96783162	T	0.070	0.054	0.945	0.331	1.30	0.76–2.21	0.056	0.054	0.020	0.888	1.02	0.76–1.37
rs469876	96785702	G	0.265	0.313	2.294	0.130	0.79	0.58–1.07	0.292	0.313	1.727	0.189	0.90	0.78–1.05
rs469783	96785820	C	0.538	0.462	4.884	0.027	1.35	1.03–1.77	0.515	0.462	9.595	0.002	1.24	1.08–1.42
rs469758	96786011	C	0.537	0.463	4.733	0.030	1.35	1.03–1.76	0.510	0.463	7.133	0.008	1.21	1.05–1.38
rs10050860	96786506	T	0.043	0.025	2.319	0.128	1.76	0.84–3.66	0.026	0.025	0.008	0.931	1.02	0.64–1.62
rs30187	96788627	T	0.533	0.459	4.801	0.028	1.35	1.03–1.76	0.506	0.459	7.662	0.006	1.21	1.06–1.38
rs26510	96790207	C	0.537	0.459	5.269	0.022	1.37	1.05–1.78	0.507	0.459	7.702	0.006	1.21	1.06–1.38
rs77311599	96793259	T	0.029	0.038	0.477	0.490	0.76	0.35–1.67	0.026	0.038	4.335	0.037	0.66	0.45–0.98
rs27434	96793809	A	0.530	0.457	4.514	0.034	1.34	1.02–1.76	0.493	0.457	4.319	0.038	1.16	1.01 to 1.32
rs2287987	96793832	C	0.067	0.055	0.629	0.428	1.25	0.72–2.15	0.056	0.055	0.025	0.875	1.02	0.76–1.38
rs998509	96797092	A	0.107	0.168	6.000	0.014	0.59	0.39–0.90	0.146	0.168	2.904	0.088	0.85	0.71–1.03
rs766222717	96797330	C	0.105	0.166	5.930	0.015	0.59	0.38–0.91	0.140	0.166	4.574	0.032	0.81	0.67–0.98
rs26653	96803547	G	0.433	0.500	3.734	0.053	0.77	0.58–1.00	0.470	0.500	2.938	0.087	0.89	0.78–1.02
rs3734016	96803761	T	0.103	0.168	6.816	0.009	0.57	0.37–0.87	0.137	0.168	6.110	0.013	0.79	0.65–0.95
rs151949	96808142	T	0.275	0.278	0.009	0.924	0.99	0.73–1.33	0.280	0.278	0.017	0.895	1.01	0.87–1.18

*Note:* Case indicates case group; Control indicates control group.

Abbreviations: Allele, allele; MAF, minimum allele frequency; SNP, single nucleotide polymorphism.

The age of onset was then divided into early‐onset (age of onset ≤ 40 years) and late‐onset PsV (age of onset > 40 years) for stratified analysis (519 early‐onset PsV and 965 healthy controls; 369 late‐onset PsV and 965 healthy controls). The results showed that among early‐onset PsV, there were eight genetic variants of *ERAP1* genetic variants associated with PsV: rs27044, rs469783, rs469758, rs30187, rs26510, rs27434, rs766222717, and rs3734016 (*p* < 0.05). Two *ERAP1* genetic variants were associated with disease in late‐onset PsV: rs27980 and rs3734016 (*p* < 0.05) (Table 7).

**TABLE 7:** Association of 18 ERAP1 variants with PsV stratified by onset age.

SNP	BP	Allele	Early‐onset psoriasis (519 cases and 965 controls)						Late‐onset psoriasis (369 cases and 965 controls)					
			MAF		*χ* ^2^	*p*	OR	95% CI	MAF		*χ* ^2^	*p*	OR	95% CI
			Case	Control					Case	Control				
rs27980	96762191	G	0.472	0.500	1.980	0.159	0.90	0.77–1.04	0.454	0.500	4.282	0.039	0.83	0.70–0.99
rs27042	96781236	A	0.251	0.272	1.463	0.226	0.90	0.76–1.07	0.283	0.272	0.373	0.541	1.06	0.88–1.28
rs27044	96783148	G	0.494	0.440	7.873	0.005	1.24	1.07–1.45	0.458	0.440	0.667	0.414	1.07	0.91–1.27
rs17482078	96783162	T	0.050	0.054	0.256	0.613	0.92	0.65–1.29	0.068	0.054	1.765	0.184	1.27	0.89–1.79
rs469876	96785702	G	0.280	0.313	3.479	0.062	0.85	0.72–1.01	0.301	0.313	0.381	0.537	0.94	0.78–1.14
rs469783	96785820	C	0.530	0.462	12.460	4.16E‐04	1.31	1.13–1.53	0.501	0.462	3.304	0.069	1.17	0.99–1.39
rs469758	96786011	C	0.524	0.463	9.694	0.002	1.28	1.09–1.49	0.499	0.463	2.621	0.105	1.15	0.97–1.37
rs10050860	96786506	T	0.027	0.025	0.049	0.826	1.06	0.64–1.76	0.030	0.025	0.477	0.490	1.22	0.70–2.13
rs30187	96788627	T	0.517	0.459	9.048	0.003	1.26	1.08–1.47	0.500	0.459	3.689	0.055	1.18	0.10–1.40
rs26510	96790207	C	0.519	0.459	9.800	0.002	1.27	1.09–1.48	0.499	0.459	3.350	0.067	1.17	0.99–1.39
rs77311599	96793259	T	0.026	0.038	2.907	0.088	0.68	0.43–1.06	0.026	0.038	2.475	0.116	0.67	0.40–1.11
rs27434	96793809	A	0.499	0.457	4.721	0.030	1.19	1.02–1.38	0.496	0.457	3.226	0.073	1.17	0.99–1.39
rs2287987	96793832	C	0.051	0.055	0.143	0.706	0.94	0.67–1.32	0.066	0.055	1.226	0.268	1.22	0.86–1.74
rs998509	96797092	A	0.144	0.168	2.941	0.086	0.83	0.67–1.03	0.137	0.168	3.694	0.055	0.79	0.62–1.01
rs766222717	96797330	C	0.135	0.166	5.113	0.024	0.78	0.63–0.97	0.136	0.166	3.752	0.053	0.79	0.62–1.00
rs26653	96803547	G	0.462	0.500	3.800	0.051	0.86	0.74–1.00	0.469	0.500	1.896	0.169	0.89	0.75–1.05
rs3734016	96803761	T	0.133	0.168	6.046	0.014	0.76	0.62–0.95	0.131	0.168	5.411	0.020	0.75	0.58–0.96
rs151949	96808142	T	0.278	0.278	0.000	0.994	1.00	0.84–1.19	0.282	0.278	0.030	0.862	1.02	0.84–1.23

*Note:* Case indicates case group; Control indicates control group.

Abbreviations: Allele, allele; MAF, minimum allele frequency; SNP, single nucleotide polymorphism.

### 3.4 Haplotype Analysis of the Association Between *ERAP1* and PsV

We examined haplotypes in the validation samples by using not only the 12 associated variants (rs27980, rs27044, rs469783, rs469758, rs30187, rs26510, rs77311599, rs27434, rs998509, rs766222717, rs26653, rs3734016) but also the four associated nonsynonymous mutation (rs27044, rs30187, rs26653, and rs3734016). The haplotype analysis of the 12 associated variants showed that a risk haplotype (TCCCTCCAGACC) was significantly associated with PsV (*p* = 0.00528, OR = 1.679 (1.03–2.74)) (Table 8). However, the haplotype analysis of the four associated nonsynonymous mutations showed that three risk haplotypes were associated with PsV (*p* = 0.000154) (Table 9). The most risk haplotype was E730/K528/R127/E56, with OR = 1.946 (1.26; 2.99). All four associated nonsynonymous mutations were highly LD with each other (Table 10).

**TABLE 8:** Haplotype analysis of 12 associated SNPs in validated stage.

Haplotypes	All samples^a^			HLA‐C*06:02 positive samples^b^			HLA‐C*06:02 negative samples^c^		
	Freq of haplotype		OR (95% CI)	Freq of haplotype		OR (95% CI)	Freq of haplotype		OR (95% CI)
	Case	Control		Case	Control		Case	Control	
GCTTCTCGGAGC	0.241	0.255	1	0.232	0.254	1	0.255	0.253	1
GGCCTCCAGACC	0.084	0.073	1.247 (0.94; 1.65)	0.080	0.089	1.018 (0.603; 1.72)	0.092	0.072	1.236 (0.84; 1.82)
TCTTCTCGGAGC	0.079	0.069	1.186 (0.883; 1.59)	0.077	0.086	0.9622 (0.567; 1.63)	0.080	0.066	1.173 (0.765; 1.8)
TGCCTCCAGACC	0.381	0.358	1.122 (0.952; 1.32)	0.401	0.341	1.298 (0.938; 1.8)	0.352	0.362	0.9707 (0.773; 1.22)
GCTTCTCGACGT	0.129	0.153	0.8483 (0.682; 1.06)	0.128	0.134	0.9908 (0.651; 1.51)	0.129	0.158	0.7862 (0.577; 1.07)
TCTTCTTGGACC	0.026	0.037	0.6994 (0.468; 1.05)	0.029	0.046	0.666 (0.346; 1.28)	0.021	0.035	0.783 (0.249; 2.46)
TCCCTCCAGACC	0.025	0.016	1.679 (1.03; 2.74)	0.022	0.017	1.359 (0.506; 3.65)	0.031	0.016	0.568 (0.304; 1.06)
TCTTCTCGACGT	0.000	0.010	—	0.000	0.000	—	0.000	0.011	1.988 (1.06; 3.73)
GCCCTCCAGACC	0.000	0.000	—	0.000	0.011	—	0.000	0.000	—

^a^ Likelihood ratio test: *χ* ^2^ = 18.4, df = 6, *p* = 0.00528.

^b^ Likelihood ratio test: *χ* ^2^ = 6, df = 6, *p* = 0.423.

^c^ Likelihood ratio test: *χ* ^2^ = 14.6, df = 7, *p* = 0.042.

**TABLE 9:** Haplotype analysis of four associated nonsynonymous mutations in validated stage.

Allotypes	Haplotypes	All samples^a^			HLA‐C*06:02 positive samples^b^			HLA‐C*06:02 negative samples^c^		
		Freq of haplotype		OR (95% CI)	Freq of haplotype		OR (95% CI)	Freq of haplotype		OR (95% CI)
		Case	Control		Case	Control		Case	Control	
E730/R528/P127/K56	CCGT	0.132	0.165	1	0.136	0.142	1	0.136	0.170	1)
E730/R528/P127/E56	CCGC	0.327	0.326	1.257 (1.03; 1.54)	0.344	0.339	1.049 (0.71; 1.55)	0.344	0.322	1.32 (0.998; 1.75)
Q730/K528/R127/E56	GTCC	0.468	0.431	1.373 (1.13; 1.66)	0.448	0.430	1.3 (0.889; 1.9)	0.448	0.433	1.291 (0.987; 1.69)
E730/K528/R127/E56	CTCC	0.035	0.022	1.946 (1.26; 2.99)	0.040	0.027	1.249 (0.55; 2.84)	0.040	0.020	2.383 (1.35; 4.2)
E730/R528/R127/E56	CCCC	0.030	0.047	0.7841 (0.536; 1.15)	0.023	0.055	0.6986 (0.362; 1.35)	0.023	0.046	0.6162 (0.339; 1.12)

^a^ Likelihood ratio test: *χ* ^2^ = 22.6, df = 4, *p* = 0.000154.

^b^ Likelihood ratio test: *χ* ^2^ = 5.56, df = 4, *p* = 0.235.

^c^ Likelihood ratio test: *χ* ^2^ = 17, df = 4, *p* = 0.00191.

**TABLE 10:** LD analysis of four associated nonsynonymous mutations (*r* ^2^/D′).

Mutations	rs27044	rs30187	rs26653	rs3734016
rs27044	1			
rs30187	0.882/0.988	1		
rs26653	0.737/0.965	0.833/0.976	1	
rs3734016	0.148/0.993	0.166/1.000	0.190/1.000	1

## 4 Discussion

Psoriasis is a common chronic, inflammatory, and relapsing disease that can be divided into PsV, erythrodermic psoriasis, pustular psoriasis, and articular psoriasis. Psoriasis is associated with a variety of common diseases, including arthritis, cardiovascular disease, obesity, hypertension, and diabetes (Zhu et al. 2013). PsV is generally recognized as a genetic disorder that is influenced by ethnic, regional, and environmental factors. Genetic studies have shown that *PSORS1* on chromosome 6p21.3 is the most significant genetic factor associated with PsV, and the possible risk allele in *PSORS1* is HLA‐C*06:02 (Dand et al. 2019). *ERAP1* is a gene associated with PsV, located on chromosome 5q15, and encodes an aminopeptidase that belongs to the M1 family of zinc‐finger metallomatrix peptidases. Recent studies have shown that the interaction of *ERAP1* with related *ERAP2* is crucially involved in PsV (Marusina et al. 2023; Zhang et al. 2022; Wu and Zhao 2021; Wiśniewski et al. 2018).

Two distinct conformational states of *ERAP1* were observed, namely, the open form and the closed form. Domain IV in the closed form interacts with domains I and II, but in one open form domain IV is oriented away from domain II. The structural difference between the two forms is attributed to the differences in the conformation of domain III, resulting in a dramatic topological change in domain IV, and the closed form of *ERAP1* creates an active catalytic pocket in the large cavity formed by domains I, II, and IV (Hanson et al. 2019). Lys528 is located on the surface of domain III, where it forms an interface between domain III and domains II and IV. Allelic alterations in s528 may affect the conformational dynamics of *ERAP1* and lead to changes in its catalytic properties. R725Q and Q730E decrease the activity of the peptide N‐terminal and *ERAP1* expression, respectively, resulting in an overall decrease in MHC class I expression (Reeves et al. 2013).

Genetic variants of *ERAP1* rs72773968 (T12I), rs3734016 (E56K), rs26653 (P127R), rs26618 (I276M), rs27895 (G346D), rs2287987 (M349V), rs30187 (K528R), rs10050860 (D575N), rs17482078 (R725Q), and rs27044 (Q730E) have been shown to be associated with ankylosing spondylitis and Behcet's disease (García‐Medel et al. 2012; Remmers et al. 2015). Haplotype K528 / Q730 has been reported to be a risk factor for PsV, while P127 and E730 are protective factors for PsV (Ombrello, Kastner, and Remmers 2015). Polymorphisms in the *ERAP1* gene may alter the conformational structure, specificity, and activity of proteins. rs17482078 (R725Q) and rs27044 (Q730E) located on the internal surface of the C‐terminal cavity may affect substrate sequence or specificity. rs26653 (R127P), rs30187 (K528R), and rs10050860 (D575N) are located in the linker region and are able to indirectly affect the specificity and activity of the enzyme (Tri, Tran, et al. 2015). In various autoimmune diseases *ERAP1* variants show interactions with MHC class I risk alleles. The interaction between HLA‐C*06:02 and *ERAP1* may affect PsV, but the specific mechanism remains unknown (Alvarez‐Navarro 2014; López de Castro 2018).

To screen significant genetic variants, we performed second‐generation sequencing of all 27 exons of *ERAP1* in 142 patients with PsV and 100 healthy controls and identified 13 significant genetic variants, including rs27980, rs27042, rs27044, rs469876, rs469783, rs469758, rs26510, rs27434, rs998509, rs766222717, rs3734016, rs151949, and rs77153199. To minimize error, a large sample size was used for second‐stage validation sequencing. The results of primary screening sequencing and validation sequencing were combined by CMH method, and we showed that the significant genetic variant rs26653_G in the validation stage was not significant in the pooled statistical analysis (*p* = 0.098), and rs469876_G, which was not significant in the validation stage, was associated with PsV in the pooled analysis (*p* = 0.013).

HLA‐C*06:02 has the strongest association with PsV and is a risk factor for the pathogenesis of PsV (Zhou et al. 2016). *ERAP1* plays a role in processing peptides for presentation to class I MHC, and HLA‐C*06:02 belongs to MHC class I molecules (Ghosh, Di Marco, and Stevanović 2019). HLA‐C*06:02 has specific peptide binding motifs and peptide presentation capacity and triggers CD8^+^ T cell‐specific immune responses. We grouped HLA‐C*06:02 negativity and positivity with the 18 selected gene variants selected for validation sequencing, and the results showed that when HLA‐C*06:02 was positive, rs469783_C, rs469758_C, and rs26510_C were associated with the onset of PsV and were risk factors for the onset of PsV. However, when HLA‐C*06:02 was negative, there was no significant correlation at the above 18 gene loci. These results suggest that *ERAP1* may produce or destroy psoriatogenic peptides for antigen presentation by HLA‐C*06:02, and different genetic variants of *ERAP1* may do it with different efficiency.

In the previous study, Fu et al. (2018) analyzed 143 cases in China and showed that the CC genotype and C allele of rs26653 were significantly associated with an increased risk of early‐onset PsV but not late‐onset PsV. It is well known that early‐onset PsV is much stronger associated with HLA‐C*06:02 than late‐onset disease. However, there was no significant difference in rs26653 early‐onset versus late‐onset PsV in our study.

Previous reports have indicated that a large proportion of patients with PsV have a family history, and the chance of developing PsV is increased in first‐ or second‐degree relatives of patients with PsV (Masouri et al. 2016). Fu et al. (2018) performed correlation analysis based on family history and showed that rs26653_CC genotype was significantly associated with increased risk of PsV in patients with family history, and there was no significant association between rs26653 genetic variants and PsV risk in patients without a family history. However, there was no significant association between rs26653 and negative or positive family history in our study. The reason may be due to the fact that Northern Han from Inner Mongolia are different from Southern Han but similar to Mongols by many molecular polymorphisms (Tian et al. 2024; Gu et al. 2022; Yu and Li 2021).

In conclusion, by two‐stage association analysis we found that 12 genetic variants of *ERAP1* gene were associated with PsV in Inner Mongolia Han nationality. HLA‐C*06:02 may interact with *ERAP1* gene and affect the risk of PsV in Inner Mongolia Han nationality. In HLA‐C*06:02 positive samples, *ERAP1* genetic variants rs469783_C, rs469758_C, and rs26510_C were associated with PsV and were risk factors for PsV. A risk haplotype of four‐nonsynonymous mutation (E730/K528/R127/E56) is associated with PsV, which might interact with HLA‐C*06:02.

## Author Contributions

X.L., J.B., L.A., F.‐R.Y., B.Y., Y.‐P.H., N.L., W.‐Y.D., Z.‐Q.S., and X.‐X.L. collected and analyzed the samples and performed data analysis. J.‐W.H. designed and supervised the study and wrote the manuscript. All authors read and approved the manuscript.

## Conflicts of Interest

The authors declare no conflicts of interest.

## Data Availability

All data are available upon request to the corresponding author.
